# The role of neurofilament light in genetic frontotemporal lobar degeneration

**DOI:** 10.1093/braincomms/fcac310

**Published:** 2022-11-26

**Authors:** Henrik Zetterberg, Charlotte Teunissen, John van Swieten, Jens Kuhle, Adam Boxer, Jonathan D Rohrer, Laura Mitic, Alexandra M Nicholson, Rodney Pearlman, Stella Mayo McCaughey, Nadine Tatton

**Affiliations:** Department of Psychiatry and Neurochemistry, University of Gothenburg, Gothenburg, Sweden; Clinical Chemistry, Sahlgrenska University Hospital, Gothenburg, Sweden; Dementia Research Institute, University College London, London, UK; DRI Fluid Biomarker Laboratory, Hong Kong Center for Neurodegenerative Diseases, Hong Kong, China; Department of Clinical Chemistry, Amsterdam University Medical Center, Vrije Universiteit Amsterdam, Amsterdam, the Netherlands; Department of Neurology, Erasmus Medical Center, Rotterdam, the Netherlands; Department of Clinical Research, Department of Neurology, Department of Biomedicine, Multiple Sclerosis Centre, University Hospital Basel, University of Basel, Basel, Switzerland; Department of Neurology, Memory and Aging Center, University of California San Francisco, San Francisco, CA, USA; Queen Square UCL Institute of Neurology, Dementia Research Centre, UK Dementia Research Institute, University College London, London, UK; Department of Neurology, Memory and Aging Center, University of California San Francisco, San Francisco, CA, USA; The Bluefield Project to Cure FTD, San Francisco, CA, USA; The Bluefield Project to Cure FTD, San Francisco, CA, USA; Department of Neuroscience, Mayo Clinic Jacksonville, Jacksonville, FL, USA; The Bluefield Project to Cure FTD, San Francisco, CA, USA; Medical Affairs, Alector, Inc., South San Francisco, CA, USA; Medical Affairs, Alector, Inc., South San Francisco, CA, USA

**Keywords:** frontotemporal lobar degeneration, neurofilament light, trial stratification, disease progression, frontotemporal dementia

## Abstract

Genetic frontotemporal lobar degeneration caused by autosomal dominant gene mutations provides an opportunity for targeted drug development in a highly complex and clinically heterogeneous dementia. These neurodegenerative disorders can affect adults in their middle years, progress quickly relative to other dementias, are uniformly fatal and have no approved disease-modifying treatments. Frontotemporal dementia, caused by mutations in the *GRN* gene which encodes the protein progranulin, is an active area of interventional drug trials that are testing multiple strategies to restore progranulin protein deficiency. These and other trials are also examining neurofilament light as a potential biomarker of disease activity and disease progression and as a therapeutic endpoint based on the assumption that cerebrospinal fluid and blood neurofilament light levels are a surrogate for neuroaxonal damage. Reports from genetic frontotemporal dementia longitudinal studies indicate that elevated concentrations of blood neurofilament light reflect disease severity and are associated with faster brain atrophy. To better inform patient stratification and treatment response in current and upcoming clinical trials, a more nuanced interpretation of neurofilament light as a biomarker of neurodegeneration is now required, one that takes into account its relationship to other pathophysiological and topographic biomarkers of disease progression from early presymptomatic to later clinically symptomatic stages.

## Teaser

Zetterberg *et al.* report that neurofilament light is a useful inclusion criterion supporting participant stratification in genetic frontotemporal lobar degeneration trials. Annualized rate of change in blood neurofilament light may distinguish frontotemporal dementia-*GRN* and -*C9orf72* mutation subtypes and provide prognostic value, especially in combination with other pathophysiological and topographic biomarkers.

## Graphical Abstract

**Graphical abstract:**
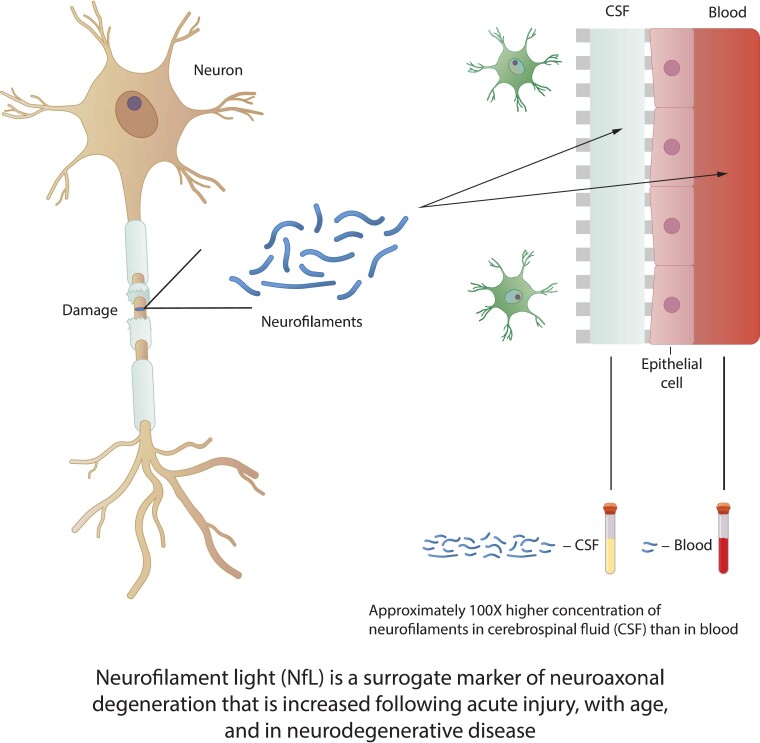

## Introduction

This review is the synthesis of a meeting convened by Alector, Inc., and the Bluefield Project to Cure Frontotemporal Dementia in December 2020. Key opinion leaders from academia with expertise in genetic frontotemporal dementia (FTD), multiple sclerosis, biomarkers and drug development, and the use of neurofilament light (NfL) as a biomarker in neurodegenerative diseases were invited speakers. Meeting attendees (list provided in manuscript addendum) represented various companies in addition to clinical and basic scientists with interest in biomarkers and drug development for neurological diseases.

Frontotemporal lobar degeneration (FTLD) underlies a group of clinically, genetically and pathologically diverse clinical syndromes involving proteinopathies that are associated with dysfunction of the frontotemporal networks. FTD refers to canonical presentations of FTLD at the symptomatic (dementia) stage. The clinical syndromes are often, but not always, associated with a progressive neurodegenerative pathology; however, prediction of the underlying pathology based on clinical presentation is problematic. These disorders are challenging to diagnose, particularly in the preclinical stage through advancement to the prodromal stage. Optimally, disease-modifying treatments should be tested at these earlier stages, while judgment is preserved, and individuals still retain their independence. Ideally, fluid and imaging biomarkers could provide information on the start of pathological changes, but in sporadic FTLD, biomarkers are currently used to support a diagnosis based on established clinical consensus criteria. In cases where there is a suggestive family history, an estimate of years to disease onset may be feasible, and genetic testing can be done to reveal a causal mutation to support the diagnosis. Fluid and imaging markers could then be employed to determine the sequence of events contributing to disease pathobiology and their correlation with clinical progression.^1^

Autosomal dominant mutations in the progranulin (*GRN*), chromosome 9 open reading frame 72 (*C9orf72*) and microtubule associated protein tau (*MAPT*) genes are recognized as the major causes of the highly heritable forms of genetic FTLD. It is estimated that these three genes are responsible for approximately half of familial FTD cases and approximately 10–30% of all FTD cases, whereas the remainder is considered sporadic.^2^ FTLD is pathologically associated primarily with either misfolded transactive response (TAR) DNA-binding protein 43 (TDP43) or misfolded tau protein aggregates in neurons. Longitudinal data from the Genetic FTD Initiative (GENFI; www.genfi.org) and Advancing Research and Treatment for Frontotemporal Lobar Degeneration/Longitudinal Evaluation of Familial FrontoTemporal Dementia Subjects (ALLFTD; www.allftd.org) networks,^3,4^ as well as a network of French amyotrophic lateral sclerosis (ALS) and FTD clinics,^5^ suggest that NfL may be a valuable blood-based biomarker, reporting that NfL baseline levels and rate of change with disease progression differ among the FTLD gene mutation subtypes as compared with healthy controls.

Several clinical trials testing experimental, potentially disease-modifying treatments are underway for FTD caused by the progranulin gene mutation (FTD-*GRN*), employing unique strategies to restore progranulin levels, including a monoclonal antibody trial to block the progranulin/sortilin pathway (NCT04374136), two separate gene therapy trials using adeno-associated viral vectors (NCT04408625; NCT04747431), and a protein replacement therapy trial (NCT05262023). Interventional trials for FTD caused by the *C9orf72* gene expansion (FTD-*C9orf72*) have also begun and include a novel antisense oligonucleotide trial (NCT04931862) and a monoclonal antibody trial (NCT03987295). Both FTD-*GRN* and FTD-*C9orf72* are characterized by a TDP43 proteinopathy. TDP43 is an RNA/DNA-binding protein involved in RNA metabolism that can aggregate in neuronal and glial cell cytoplasm.^6^ It is assumed that increases in blood NfL levels may signal an undesirable change based on its steady increase with aging, as well as its elevation in acute injury and chronic neurological diseases when compared with healthy control populations.^7,8^ Interpretation of short-term changes in absolute levels of blood-based NfL in healthy populations and clinical trials of disease-modifying therapies for FTLD disorders is an emerging need. Longitudinal NfL dynamics relative to the time course of structural changes in FTLD to improve our comprehension of advancing neurodegeneration through the presymptomatic, proximity to conversion (prodromal), and clinically symptomatic stages of genetic FTLD are also needed, in addition to other pathophysiological markers. Additionally, a better understanding is required of NfL dynamics and turnover (e.g. the potential role of microglia in NfL clearance) and the relationship between NfL and comorbidities such as subclinical brain injuries, cardiovascular events, diabetes, and prescribed medication use, which can all increase blood-based NfL levels.^9,10^

## Axonal damage after acute injury and in chronic neurodegenerative disease

Neurofilaments (NFs) are class IV intermediate filaments composed of three subunits of different molecular sizes: 68 kDa NfL, 160 kDa neurofilament medium (NFM), and 200 kDa neurofilament heavy (NFH), combined with α-internexin as the fourth subunit in CNS axons, and function as one of the cytoskeletal elements of neurons.^11^ NFs are released into the extracellular space following axonal injury and have been hypothesized to act as surrogate markers of neurodegeneration. Release of NFs, particularly NfL and NFH, into the CSF, and eventually the bloodstream is proposed to occur after axotomy and secondary axonal injury.^11,12^ Some adult animal models show NfL accumulation before NFM and NFH, indicating an organized, temporal sequence of events postinjury.^13^ Case studies in athletes have demonstrated that elevated NfL levels can persist at least 30 weeks in the CSF following some acute injuries,^14,15^ arguing that absolute NfL levels, as well as duration of elevation, may reflect continuous axonal injury. An examination of acute, mild traumatic brain injury cases suggests that the delayed peak in plasma NfL levels after injury may reflect ongoing secondary injury or repair mechanisms.^16^

Post-mortem studies in ALS revealed that degenerating neurons had a 70% decrease in NfL mRNA, as well as decreases in α-internexin and peripherin.^17^ TDP43, the pathognomonic intracellular protein aggregate common to ALS (*C9orf72*) and FTD (*C9orf72, GRN)*, is able to bind and destabilize, or sequester, neurofilament light gene (*NEFL*) mRNA by direct interaction with the three prime untranslated region.^18,19^ This has implications for altered RNA processing in ALS, which can ultimately impact NfL protein synthesis, stoichiometry, and aggregation.^20^ Pathology studies have elucidated the trajectory of nerve cell degeneration in chronic neurodegenerative diseases, whereby misfolded proteins like TDP43 spread through a disease-specific neural connectome and aggregate in distinct neuronal populations, ultimately resulting in functional deficits and death.^21^ It is not known whether specific messenger RNA-binding proteins that normally regulate *NEFL* RNA processing, trafficking, and local protein synthesis within axons and dendrites play an active role in chronic neurodegenerative diseases or secondary axonal injury.^22^ Animal models have demonstrated that the specialized transcriptome in axons allows them to quickly alter the axonal proteome via local translation and protein degradation.^23–25^ This capacity for local translation in axons can provide cells with the ability to respond to external cues that impact axon elongation, synaptic function, and maintenance during development and adulthood.^26,27^

## NfL as a biomarker of disease progression and drug efficacy in multiple sclerosis

Our most comprehensive understanding to date of how NfL may be implemented as a disease activity/progression marker and disease treatment efficacy marker comes from multiple sclerosis studies. Multiple sclerosis has a strong neuroaxonal injury component, which is the likely substrate of the observed clinical disability. The neuropathology includes axonal bulbs, transection, and demyelination of the axon,^28^ with acute axonal degeneration appearing early in the disease course and most prominently within the first year after disease onset.^29^ Chronic spinal plaques in multiple sclerosis reflect 58–68% axonal loss.^30^ Although several anti-inflammatory treatments are available for relapsing multiple sclerosis, there is a struggle to understand if treatments suppress subclinical, ‘benign’ multiple sclerosis.^31^ A precision medicine approach would be preferred to treat individuals, incorporating biomarkers as well as companion treatments that would lead to a demonstrable halt or slowed rate of neurodegeneration. Patients with progressive or relapsing multiple sclerosis show higher serum NfL levels than healthy controls, and the correlation is strong between CSF and serum NfL, with disease-modifying treatments demonstrating similar effect on NfL in both fluids.^32^ Recent trials in progressive multiple sclerosis also demonstrate that increased NfL levels are associated with ongoing disease activity, as well as long-term outcome.^33^ A multisite, longitudinal study involving >10 000 serum samples from >5000 participants without evidence of CNS disease spanning over six decades of life is underway in an effort to generate age-adjusted reference values for NfL.^34^ When compared with reliable, age-adjusted reference values, blood-based NfL may be considered a first-in-class biomarker for multiple sclerosis disease activity with prognostic value,^35^ and some hope for a potential clinical application. Short-term neuroprotective studies using area under the curve analysis of serum NfL levels as an outcome for screening effective neuroprotective drugs for late phase 3 clinical development may prove a valuable concept. A Letter of Support was granted to the International Progressive multiple sclerosis Alliance (https://www.progressivemsalliance.org/) by the US Food and Drug Administration (FDA; https://www.fda.gov/media/149608/download) in June 2021 to encourage further study and use of NfL in plasma or serum as a pharmacodynamic/response biomarker for early clinical trials in progressive multiple sclerosis.

## Multidecade and individual variability of NfL over time in aging and dementia cohorts

Data collected every five years in the 30-year Betula study in Sweden reported that age-related changes in plasma NfL levels (measured by Simoa) are linked to brain white matter (WM) alterations using T1- and T2-weighted images.^36^ Alzheimer’s disease cases showed elevated levels compared with controls, but no significant differences from the preclinical phase. NfL did not predict age-related cognitive impairment or impending Alzheimer’s disease. By contrast, the Dominantly Inherited Alzheimer Network modelled that serum NfL was elevated in the presymptomatic stages of familial Alzheimer’s disease; specifically, longitudinal, within-person analysis showed that the rate of change of NfL discriminated mutation carriers from non-mutation carriers almost 10 years earlier than cross-sectional, absolute NfL levels.^37^

The single-site Austrian Stroke Prevention Study monitored a cohort of 335 adults with no obvious neurological disorder ranging in age from approximately 38 to 85 years over an approximately six-year period for serum NfL changes using a single-molecule array (Simoa assay) with UmanDiagnostics anti-core domain antibodies.^38^ Although median NfL levels were relatively similar among individuals in the fourth and fifth decades of this normal aging cohort, NfL rose non-linearly and demonstrated more variability in participants aged 60 and older. These data support the hypothesis that NfL increases with age, and those age-related degenerative processes may also contribute to NfL increase in the absence of overt clinical symptoms. Subclinical brain pathology with volume loss was supported by longitudinal and cross-sectional T1-weighted MRI of brain volume in participants and showed a close correlation with serum NfL levels.

A survey of the Alzheimer Center Amsterdam clinics revealed that clinicians perceive serum NfL to be more useful in patients younger than 60 years to confirm or exclude neurodegenerative disease versus a psychiatric diagnosis.^39^ As a result, NfL measures are now being requested to support an FTD diagnosis in this age group. A Shiny app has been developed to provide a quick comparison between disease readout and age-dependent reference values (https://networkinstitute.org/2020/11/23/neurofilament-light-application-online).

Across sporadic and genetic FTD, CSF NfL levels are higher compared to patients with Alzheimer’s disease or controls.^7,8,40^ Serum NfL levels follow a similar pattern to CSF, with FTD-*GRN* reported as higher on average (61.5 pg/ml) compared with FTD-*C9orf72* (33.9 pg/ml). However, variation can be considerable in absolute blood NfL levels within all genetic FTD groups, as well as in control subjects. On occasion, non-carriers may have a higher baseline serum NfL than a presymptomatic or symptomatic carrier. As higher NfL levels decrease to normal in these few cases, repeated measurements over time may help to interpret these outliers.^41^ In the short term, examining cohorts for variations between consecutive measurements of blood NfL levels will be valuable to understand what constitutes a clinically meaningful variation in untreated patients, patients undergoing treatment as part of their clinical care and management, and participants in clinical trials. One such study analyzed serum NfL at baseline and at six-month follow-up using the Simoa NF-Light kit in neurologically healthy controls, untreated relapsing multiple sclerosis, and secondary and primary progressive multiple sclerosis.^42^ Elevation of NfL in healthy controls was positively associated with increased age, and at follow-up, NfL was elevated compared with baseline. Baseline NfL was elevated in multiple sclerosis compared with healthy controls but was lower at follow-up in both treated and untreated relapsing multiple sclerosis. The authors concluded that variations between two consecutive measurements within individuals occur to a similar extent in healthy controls and subjects with multiple sclerosis.

## Brain structural changes, NfL, and cognition in Alzheimer’s disease dementia

There is a need to better understand NfL regulation in health and disease, particularly the relationship between NfL and cognitive decline, to support recommendations for the use of NfL as a surrogate biomarker in FTLD clinical trials. A scoping review of neurological disease literature revealed that higher levels of NfL (CSF, plasma, or serum) were generally associated with decreased cognition.^43^ However, inconsistencies in the relationship between elevated NfL and declining Mini-Mental State Examination performance were found within and across Alzheimer’s disease and other dementias. There may be dynamic relationships among cognitive performance, structural changes in the cortex and NfL levels obtained from blood and CSF, which may vary over the course of different dementias. There is evidence to suggest that mean diffusivity (MD) can evaluate changes in grey matter (GM) at a microscopic level, reflecting neuronal loss and the breakdown of myelin, cell membranes, and organelles, which results in a measurable difference in the diffusion of water molecules. MD, a metric of diffusion weighted imaging, assesses diffusion in all directions and is therefore uniquely suited to measuring microstructural change in GM.^44^ Two studies that examined cortical thickness and cortical MD in autosomal dominant Alzheimer’s disease reported a biphasic trajectory of macro- and microstructural changes.^45,46^ A close association was found between increasing MD and decreasing cortical thickness, suggesting that MD as a marker of microlevel cortical integrity may be part of the same disease pathology continuum as macrolevel neurodegeneration.^45^ Importantly, increased cortical thickness and decreased cortical MD were observed about 15–20 years prior to estimated symptom onset, followed by cortical thinning and increased cortical MD in later prodromal and symptomatic stages of autosomal dominant Alzheimer’s disease.^46^ In a separate study, NfL in the CSF was measured in 221 participants of the Australian Imaging, Biomarkers & Lifestyle Flagship Study of Ageing and was found to be significantly elevated in Alzheimer’s disease compared to healthy controls. Further, NfL levels predicted baseline cognition and cortical GM volume and were significantly correlated with tau pathology assessed by CSF total-tau and phosphorylated-tau.^47^

## Neurodegeneration associated with FTD-*GRN* and FTD-*C9orf72*

Mutations in the *GRN* gene produce haploinsufficiency, resulting in an approximately 50% decrease in progranulin protein levels and, ultimately, aggregation of misfolded TDP43 protein in vulnerable neurons. Blood progranulin levels are significantly reduced in pathogenic *GRN* mutation carriers compared with non-mutation carrier family members from childhood through adulthood in presymptomatic FTD-*GRN*. Thus, blood progranulin level provides a sensitive diagnostic biomarker, but does not appear to have a direct correlation with progressive brain atrophy.^48^

Age of onset is highly variable in FTD-*GRN*, ranging from 25 to 90 years, with a mean age of symptom onset of 61 ± 8.8 years and a mean disease duration of 7.1 years (*n* = 548).^49^*GRN* mutations appear to be nearly 100% penetrant by 80 years of age.^50^ WM hyperintensities have been associated with FTD-*GRN*^51,52^ but not with FTD-*C9orf72*. Neuropathological findings suggest that these WM hyperintensities are associated with microglial activation and microglial dystrophy.^53^ GM atrophy and hypometabolism have been reported about 10–15 years prior to clinical symptom onset.^54–57^

FTD-*C9orf72* is the most common genetic form of FTD in North America and Europe, responsible for approximately 13% of all FTD cases and approximately 11% of all ALS (ALS-*C9orf72*) cases; it can also cause a combination of ALS and FTD.^58^ FTD-*C9orf72* results from a hexanucleotide repeat expansion within the *C9orf72* gene, and there is compelling evidence to support several different mechanisms that cause this repeat to result in TDP43 neuropathology and degeneration. Symptom onset can range from 20 to 90 years of age and the mean age of onset of FTD-*C9orf72* is 58.2 ± 9.8 years, with a mean disease duration of 6.4 years (*n* = 618).^49^ The pathogenic expansion is almost fully penetrant by 80 years of age.^59^ Brain atrophy in FTD-*C9orf72* carriers occurs earlier than in other mutation groups, appearing before age 40 and potentially 25 years before clinical symptoms.^60^

Structural imaging studies have demonstrated cortical thinning in presymptomatic FTD*-GRN* carriers compared with non-carriers.^61,62^ A recent study comparing *GRN* carriers and non-carriers reported age-related cortical thinning in both groups but with significantly greater thinning in the presymptomatic *GRN* carriers.^63^ Presymptomatic *C9orf72* carriers also demonstrated greater cortical thinning compared with controls.^64^ NfL has been considered to reflect WM changes, as NFs are primarily found in axons.^65,66^ Longitudinal GM and WM changes were analyzed in presymptomatic *GRN* and *C9orf72* carriers in GENFI cohorts over 2 years, and although *C9orf72* carriers showed lower GM volume in the cerebellum and insula and WM differences in the anterior thalamic radiation compared with non-carriers, presymptomatic *GRN* carriers did not show presymptomatic GM or WM degeneration.^67^ Of interest is a study that used diffusion tensor imaging to provide evidence of a correlation between plasma NfL in behavioural variant FTD (bvFTD) and WM changes.^68^ Although it was a small study of 20 subjects, a reduction in fractional anisotropy was associated with increased plasma NfL concentration, an elevated CDR® plus NACC FTLD score, and a global level of degeneration in WM tracts that included the superior longitudinal fasciculus, the fronto-occipital fasciculus, the anterior thalamic radiation, and the dorsal cingulum bundle.

Cortical macrostructural changes, as measured by voxel-based morphometry or cortical thickness with surface-based analysis, have been the standard for neuroimaging in FTD. However, a recent multicentre study examined cortical MD and cortical thickness in a bvFTD cohort that included possible and probable bvFTD subgroups. In the whole bvFTD group, cortical MD and cortical thickness correlated with the CDR® plus NACC FTLD; but in the possible bvFTD group, only cortical MD correlated with the CDR rating scale, suggesting that cortical MD may be a more sensitive biomarker for microstructural neurodegenerative changes that can occur earlier than macrostructural changes in disease trajectory. Only minimal cortical thinning was found in the possible bvFTD subgroup compared to controls. In the whole bvFTD group, cortical thickness and cortical MD both correlated with measures of disease severity and CSF biomarkers, but the areas of correlation with cortical MD were more extensive. CSF soluble peptide amyloid precursor protein beta (sAPPβ), CSF NfL, and CDR measures had a more widespread correlation with cortical MD than cortical thickness.^69^ Cortical MD was also found to be increased in mild primary progressive aphasia with *GRN* mutation, while only minimal cortical thinning was observed. Together, these data suggest that cortical MD may be more sensitive than cortical thickness in the detection of the earliest neurodegenerative changes in FTLD and that a biphasic trajectory of neurodegeneration should be considered.^70^

Data from the GENFI network revealed a relationship between NfL and executive function, rather than cognitive performance, in FTD.^71^ And a recent case-control study supports a relationship between elevated CSF NfL in FTLD with known TDP43 pathology and a longitudinal decline in specific executive and language measures compared with controls. Further, it was shown that patients with FTLD-TDP pathology had significantly greater CSF NfL than those with FTLD-tau pathology.^72^ Longitudinal studies of genetic FTLD that include pathophysiological markers (such as NfL in CSF and blood) combined with topographical markers of neurodegeneration are needed to better understand the relationship between NfL and FTLD. In particular, examination of presymptomatic, prodromal, and symptomatic gene mutation carriers could indicate the presence of a biphasic trajectory of neurodegenerative change in FTLD, inform on absolute cutoffs between stages, and reveal potential differences in disease trajectory across the different autosomal dominant gene mutations. Longitudinal studies could also provide insight into the dynamic relationship between NfL and other biomarkers, their correlation with clinical status, and what combinations of biomarkers might improve the prognostic value of NfL over the course of these complex, highly heterogeneous FTLD disorders.

## NfL rate of change in FTD-*GRN* versus FTD-*C9orf72*

In the GENFI and ALLFTD cohorts, the conversion (prodromal) period from clinically presymptomatic to symptomatic status is accompanied by the highest rate of change in serum NfL levels at any time during the disease, and *GRN* mutation carriers demonstrate a higher rate of NfL change than FTD-*C9orf72* mutation carriers. Higher concentrations of serum NfL reflect disease severity, as they are associated with faster rates of brain atrophy, and baseline NfL was significantly elevated in subjects who converted from presymptomatic to symptomatic or showed disease progression versus non-progressors. Overall, baseline plasma NfL was highly predictive of change in clinical status over the following 2 years. Furthermore, higher baseline NfL correlated with worse longitudinal clinical measures and brain atrophy in all FTD genetic mutation types, arguing that blood-based NfL is predictive of short-term risk for FTD disease progression.^3,4,73,74^

Longitudinal changes in plasma NfL in presymptomatic and symptomatic *GRN* and *C9orf72* mutation carriers and controls (aged 21–83 years) were measured in cohorts from the French Research Network on FTD/FTD-ALS (Inserm RBM02–59), Predict to Prevent Frontotemporal Lobar Degeneration and ALS (PREV-DEMALS), and Predict-PGRN.^5^ Subjects were followed for more than 2 years, and the mean annualized rate of change (ARC) of plasma NfL was calculated along with absolute levels of plasma NfL at baseline and follow-up. *GRN* carriers’ baseline NfL was 86.21 pg/ml versus 39.49 pg/ml for *C9orf72* (*P* = 0.014) and demonstrated greater progression on ARC (+29.3%) compared with *C9orf72* carriers (+24.7%; *P* = 0.016). Of interest, ARC differed with phenotype in *C9orf72* carriers: ALS had a mean ARC of +37%, FTD was +21.7%, and psychiatric presentations were +8.3%. However, slow-progressing *C9orf72* carriers had an ARC of +2.5% compared to *C9orf72* patients with standard progression. *GRN* carriers did not display such differences, possibly because of less variability in *GRN* disease progression. Separate cutoff thresholds of 19.00 pg/ml and 27.48 pg/ml for *C9orf72* and *GRN* carriers, respectively, differentiated them from controls. In the ALLFTD cohorts, plasma NfL discriminated the presymptomatic from symptomatic mutation carriers, with a cutoff of 13.6 pg/ml in the original cohort and 19.8 pg/ml in the validation group.^3^ Data from these international cohorts demonstrate that neurodegeneration in genetic FTLD, as measured by blood NfL, has the potential to reflect disease progression and that the specific FTD gene mutation may dictate recommended cutoff thresholds.

## NfL as a pharmacodynamic marker in genetic FTLD

NfL as a pharmacodynamic marker may provide a means to distinguish treatment responders in clinical trials and, potentially, a way to gauge fast versus slow disease progressors.^5,75–77^ An individual’s disease status, as determined via blood NfL and NFH,^4^ may provide added value when combined with macro- and microstructural imaging of regional neurodegenerative changes in the brain, potentially offering greater sensitivity to earlier neurodegenerative changes in presymptomatic and prodromal stage carriers. However, longitudinal studies are required to reveal absolute levels or rate of change in NfL in relation to the trajectory of neurodegeneration measured by MD and cortical thickness neuroimaging methods. To use NfL as a pharmacodynamic marker, detailed knowledge of how the drug treatment itself influences NfL turnover is needed. Preliminary, unpublished observations suggest that NfL turnover in extracellular fluids may be influenced by microglial activity; this potential confounder would be particularly important to examine for drugs affecting microglial activation.

When compared with the apparently more gradual changes in brain GM or WM as measured with current neuroimaging methods, a fluid biomarker can provide an earlier and more accessible dynamic readout in trials that may suggest movement towards clinical improvement, to be supported later by clinical measures at 6–12 months that would reveal whether the change is statistically significant. Currently, blood-based NfL levels are supporting decisions about proceeding from investigational phase 2 studies to phase 3 trials. NfL in genetic FTLD could potentially be a more sensitive biomarker of change, but more data are needed to determine whether blood-based NfL, in combination with one or more markers, could provide greater confidence in moving forward to a pivotal trial. Longitudinal fluid biomarkers reflecting changes in the autophagy-lysosome pathway,^78^ microglial^79^ and astroglial activation markers,^80–83^ and potentially TDP43 burden,^84^ could provide additional insights into the pathobiology of FTLD and status of disease progression. Identifying the right combination of biomarkers that elucidates and refines our understanding of NfL is a work in progress, as different exploratory fluid markers are being tested in interventional trials enrolling presymptomatic and symptomatic *GRN* and *C9orf72* mutation carriers.^85^ However, such exploratory markers must also be tested in longitudinal studies in larger multicentre research cohorts and compared against healthy controls to establish their progression. For phase 3 studies, surrogacy of NfL is not yet defined, and the dynamic range of blood NfL across FTD genotypes will require absolute cutoff levels and/or rates of change defined for each FTD gene mutation population. It remains to be determined whether normalization to prodromal blood NfL ranges in genetic FTD, or prevention of further increases in absolute levels of blood NfL (or rate of change), are possible. Hopefully, the time course of NfL changes after treatment initiation compared with controls will be revealed by ongoing interventional drug trials.^3,77^

In testing NfL as a pharmacodynamic marker in genetic FTD treatment trials, the percentage change indicating clinically meaningful NfL remains unknown. The current approach is to determine if the observed changes are statistically significant in the cohort studied, but there is a need to identify and establish disease-specific cutoff points for NfL levels, or even gene mutation–specific cutoff points. In other diseases, it is known that normalization or close-to-baseline decrease in NfL is possible with treatment, but the time lag between treatment initiation and time to stable decline of NfL appears highly variable among diseases and treatment modalities when considering antisense oligonucleotide, nusinersen-treated peadiatric spinal muscular atrophy versus treated adolescents or adults,^86–88^ heamatopoietic stem cell–transplanted X-linked adrenoleukodystrophy,^89^ antiviral-treated adult HIV dementia,^90–92^ or monoclonal antibody-treated adult multiple sclerosis.^32,93^

FTD-*GRN*, the most rapidly progressing of the FTD autosomal dominant gene mutations, show the highest rate of change in blood NfL moving from presymptomatic to symptomatic clinical status, as well as the highest median baseline. NfL may help define a window of opportunity for enrolling participants into investigational disease-modifying drug trials by employing an absolute, elevated NfL concentration as a cutoff that reflects transition from presymptomatic to prodromal stage. Longitudinal data suggest that the rate of change in blood NfL from conversion stage to symptomatic stage may be of prognostic value in gauging the rate of disease progression or drug efficacy.^3,4^

NfL levels observed over the first 12 months of an investigational treatment trial for FTD-*GRN* reported that both CSF and plasma NfL levels remained stable over this time period, reflecting a consistent trend in response to experimental drug treatments.^94^ Decreased levels of blood or CSF NfL in other treatment trials have shown that a downward trend toward baseline values can take 12–24 months.^35^ Many important questions remain regarding the use of NfL as a biomarker in genetic FTD and ongoing international longitudinal studies, as well as new investigational treatment trials, are anticipated to provide new insights. At present, we may find some answers regarding trends in NfL from observational studies in neurologically healthy aging populations and treatment trials in multiple sclerosis. The Foundation for the National Institutes of Health Biomarkers Consortium Neurofilament Project is spearheading a collaborative public-private partnership effort to examine and compare available NfL assays and platforms. A goal of this partnership is to work toward a global calibration of assay readouts and to prepare an NfL biomarker qualification letter of intent to be submitted to the FDA and European Medicines Agency (https://fnih.org/what-we-do/biomarkers-consortium).

## Summary and recommendations on use of NfL as a biomarker in genetic FTLD

Present data suggest that NfL can serve as a biomarker for onset and intensity of neurodegeneration in genetic FTLD. Given that it is a general biomarker for neurodegeneration, its diagnostic capacity is limited; however, this limitation does not apply to genetic FTLD, as the genotype gives the diagnosis. In clinical trials of disease-modifying treatments, the direction of change in fluid biomarker levels remains to be discovered. Lowering and/or stabilization of NfL in genetic FTD would be an anticipated outcome if the treatment were to slow or stop neurodegeneration in the brain. Longitudinal FTLD studies that combine pathophysiological markers with sensitive topographic biomarkers correlated with established clinical instruments to reflect disease progression are required. The timing and effect size of NfL change is difficult to address. Here, stabilization and/or lowering of NfL to levels seen in same-age healthy controls, such as those observed in patients with multiple sclerosis under highly effective treatments, may predict clinical improvement. Considering the heterogeneity of genetic and sporadic FTLD, it would be advisable to combine NfL with other pathophysiological markers to provide a more robust readout of disease progression. Lysosomal/endosomal processing, microglial and astroglial activation, and potentially changes in total-tau and phosphorylated-tau protein may help identify possible inflection points moving from presymptomatic to prodromal to symptomatic stages.^95^ In clinical trials, NfL can be used as both an inclusion criterion to ascertain that the patient has entered the neurodegenerative stage of the disease, and a prognostic biomarker. NfL in combination with other markers could potentially offer more precision on disease trajectory and interpretation of disease-modifying treatments in clinical trials. Cutoff criteria for elevated NfL levels in FTD-*GRN* and FTD-*C9orf72* should now be established. Most data suggest that CSF and plasma/serum NfL levels and rates of change are higher in *GRN* carriers but may otherwise give similar information in trials across the genetic FTLD spectrum. Plasma NfL levels may provide additional value as these levels relate to patient stratification in trials. Disclosing NfL status to patients with FTD can be complicated, since trial inclusion of participants with elevated NfL implies that their disease status has progressed (although additional markers are likely needed to establish an individual’s disease progression). Conversely, exclusion of patients from a trial because of ‘low’NfL levels may result in a difficult conversation with the prospective participants because they have a disease that progresses quickly and will ultimately take away their independence.

## Supplementary Material

fcac310_Supplementary_Data

## Data Availability

The names and affiliations of the Neurofilament task force participants is provided in Supplementary_Data-docx.

## Appendix I

### NfL Task Force Participants

Anne Alward, Anthony Bannon, Martina Bocchetta, Joshua Caufield, Irene Y. Choi, Tania F. Gendron, Danielle L. Graham, Fen Huang, Serena Hung, Gerhard Koenig, Joel A. Mathews, Michelle I. Mighdoll, Laurence Mignon, Thomas Misko, Glenn Morrison, Yan G. Ni, Robert Paul, Leonard Petrucelli, Rob Plasschaert, Maria S. Quinton, Harro Seelaar, Arthur Simen, Aitana Sogorb-Esteve, Ina Tesseur, Maria Tome, Olga Uspenskaya, Emma L. van der Ende, Mike Ward, Felix L. Yeh (further details in Supplementary material).

## Abbreviations

ALLFTD =: advancing research and treatment for frontotemporal lobar degeneration/longitudinal evaluation of familial frontotemporal dementia subjects
ALS =: amyotrophic lateral sclerosis
ARC =: annualized rate of change
bvFTD =: behavioural variant frontotemporal dementia
*C9orf72* =: chromosome 9 open reading frame 72 gene
FDA =: US Food and Drug Administration
FTD =: frontotemporal dementia
FTD-*C9orf72* =: frontotemporal dementia caused by the *C9orf72* gene mutation
FTD-*GRN* =: frontotemporal dementia caused by the progranulin gene mutation
FTLD =: frontotemporal lobar degeneration
GENFI =: genetic frontotemporal dementia initiative
GM =: grey matter
*GRN* =: granulin gene
m =: messenger
*MAPT* =: microtubule-associated protein tau gene
MD =: mean diffusivity
*NEFL* =: neurofilament light gene
NF =: neurofilament
NFH =: neurofilament heavy
NfL =: neurofilament light
NFM =: neurofilament medium
sAPPβ=: soluble peptide amyloid precursor protein beta
TDP43 =: transactive response DNA-binding protein 43
WM =: white matter
