# Supplementary material for: The role of neurofilament light in genetic frontotemporal lobar degeneration
Source: Brain Commun. 2022 Nov 26;5(1):fcac310. doi: 10.1093/braincomms/fcac310 (PMC9866262; doi:10.1093/braincomms/fcac310)
Supplement: fcac310_Supplementary_Data [file fcac310_supplementary_data.docx]

**NFL Task Force Participants**

Anne Alward, Bluefield Project (USA), San Francisco, California, United States

Anthony Bannon, PhD, AbbVie (USA), North Chicago, Illinois, United States

Martina Bocchetta, PhD, Dementia Research Centre, Department of Neurodegenerative Disease, UCL Queen Square Institute of Neurology, University College London, London, United Kingdom

Joshua Caufield, Alector (USA), South San Francisco, California, United States

Irene Y. Choi, PhD, Verge Genomics (USA), South San Francisco, California, United States

Tania F. Gendron, PhD, Mayo Clinic (USA), Jacksonville, Florida, United States

Danielle L. Graham, PhD, Biogen (USA), Cambridge, Massachusetts, United States

Fen Huang, PhD, Denali Therapeutics (USA), South San Francisco, California, United States

Serena Hung, MD, Arkuda (USA), Watertown, Massachusetts, United States

Gerhard Koenig, PhD, Arkuda (USA), Watertown, Massachusetts, United States

Joel A. Mathews, PhD, Ionis (USA), Carlsbad, California, United States

Michelle I. Mighdoll, Ms, MBA, Verge Genomics (USA), South San Francisco, California, United States

Laurence Mignon, PhD, Ionis (USA), San Diego, California, United States

Thomas Misko, PhD, AbbVie (USA), Chicago, Illinois, United States

Glenn Morrison, PhD, Alector (USA), South San Francisco, California, United States

Yan G. Ni, PhD, Passage Bio (USA), Philadelphia, Pennsylvania, United States

Robert Paul, MD, PhD, Alector (USA), South San Francisco, California, United States

Leonard Petrucelli, PhD, Mayo Clinic (USA), Jacksonville, Florida, United States

Rob Plasschaert, PhD, AVROBIO (USA), Cambridge, Massachusetts, United States

Maria S. Quinton, PhD, Takeda (USA), Cambridge, Massachusetts, United States

Harro Seelaar, MD, PhD, Erasmus University Medical Center, Rotterdam, the Netherlands

Arthur Simen, MD, PhD, Takeda (USA), Cambridge, Massachusetts, United States

Aitana Sogorb-Esteve, PhD, Dementia Research Institute at University College London, UCL Queen Square Institute of Neurology, University College London, London, United Kingdom; Dementia Research Centre, Department of Neurodegenerative Disease, UCL Queen Square Institute of Neurology, University College London, London, United Kingdom

Ina Tesseur, PhD, UCB Biopharma SRL (Belgium), Braine-l’Alleud, Belgium

Maria Tome, MASc, MD, PhD, FFPM, EMA (Europe), Amsterdam, the Netherlands

Olga Uspenskaya, MD, PhD, Prevail (USA), Paris, France

Emma L. van der Ende, MD, Erasmus University Medical Center, Rotterdam, the Netherlands

Mike Ward, PhD, Alector (USA), South San Francisco, California, United States

Felix L. Yeh, PhD, Alector (USA), South San Francisco, California, United States
